# Efficacy of a new membrane obturator prosthesis in terms of speech, swallowing, and the quality of life of patients with acquired soft palate defects: study protocol of the VELOMEMBRANE randomized crossover trial

**DOI:** 10.1186/s13063-022-06163-6

**Published:** 2022-03-18

**Authors:** Adrien Naveau, Marion Kret, Valérie Plaire, Olivier Delorme, Sébastien Marchi, Caroline de Bataille, Florent Destruhaut, Elise Arrive, Christophe Bou

**Affiliations:** 1 CHU de Bordeaux, Pôle de Médecine et Chirurgie Bucco-Dentaire, 33000 Bordeaux, France; 2 Université de Bordeaux, UFR des Sciences Odontologiques, 33076 Bordeaux, France; 3 INSERM, Bio-ingénierie Tissulaire BioTisU1026, 33076 Bordeaux Cedex, France; 4 CHU de Bordeaux, USMR, Pôle Santé publique, 33076 Bordeaux, France; 5 CHU de Bordeaux, DRCI, 33076 Bordeaux, France; 6 Université de Toulouse, Faculté de Chirurgie Dentaire, 31062 Toulouse, France; 7 CHU de Toulouse, Hôpital de Rangueil, 31059 Toulouse Cedex, France; 8 Evolution and Oral Health Laboratory (EvolSan), Paul Sabatier University, Toulouse, France

**Keywords:** Randomized controlled trial, Palatal obturator, Mouth neoplasm, Velopharyngeal insufficiency, Speech disorder, Deglutition disorder, Maxillofacial prosthesis, Prosthodontics

## Abstract

**Background:**

Soft palate defects created during oral cancer surgery may prevent complete palatal closure and trigger palatopharyngeal insufficiency. One current treatment employs a rigid obturator prosthesis; an extension of acrylic resin at the level of the hard palate ensures surface contact with the remaining musculature. Unfortunately, airflow escape often causes hypernasality, compromises speech intelligibility, and creates swallowing problems (including leakage of food and fluid into the nasal airway). We plan to test a new removable denture featuring a thick dental dam that serves as a membrane obturator. The principal objective of the clinical trial is a comparison of speech handicap levels after 1 month in patients with acquired velar insufficiencies who wear either the new device or a conventional, rigid obturator. The secondary objectives are between-device comparisons of the swallowing handicaps and the health-related qualities of life.

**Methods:**

The VELOMEMBRANE trial is a superiority, open-labeled, two-way, random crossover clinical trial. Adult patients exhibiting velar or palatovelar substance loss after tumor excision and who are indicated for rigid obturator-mediated prosthetic rehabilitation will be recruited in two teaching hospitals in France. Fourteen participants will be randomly allocated to wear both prostheses for 1-month periods in either order. The new membrane obturator is a removable resin prosthesis incorporating a rigid extension that holds a dental dam to restore the soft palate. The primary outcome will be the extent of phonation-related disability (the overall score on the Voice Handicap Index [VHI]). The secondary outcomes will be the Deglutition Handicap Index and health-related quality of life scores of the European Organization for Research and Treatment of Cancer (EORTC).

**Discussion:**

High-quality evidence will be provided to document the utility of a new medical device that may greatly improve the management and quality of life of patients with acquired velar insufficiency.

**Trial registration:**

ClinicalTrials.govNCT04009811. Registered on 4 July 2019

**Supplementary Information:**

The online version contains supplementary material available at 10.1186/s13063-022-06163-6.

## Administrative information

**Table:** Note: the numbers in curly brackets in this protocol refer to SPIRIT checklist item numbers. The order of the items has been modified to group similar items (see http://www.equator-network.org/reporting-guidelines/spirit-2013-statement-defining-standard-protocol-items-for-clinical-trials/).

Title {1}	EFFICACY OF A NEW MEMBRANE OBTURATOR PROSTHESIS IN TERMS OF SPEECH, SWALLOWING, AND THE QUALITY OF LIFE OF PATIENTS WITH ACQUIRED SOFT PALATE DEFECTS: STUDY PROTOCOL OF THE VELOMEMBRANE RANDOMIZED CROSSOVER TRIAL
Trial registration {2a and 2b}.	ClinicalTrials.gov; identifier NCT04009811. Registered on 4 July 2019
Protocol version {3}	Protocol version 1.0 (July 2019)
Funding {4}	Grant from the Bordeaux University Hospital via an internal clinical research program (Appel D’offre Interne, CHUBX 2018/34).
Author details {5a}	Adrien Naveau^1-3^, Marion Kret^4^, Valérie Plaire^1^, Olivier Delorme^5^, Sébastien Marchi^5^, Caroline de Bataille^6-7^, Florent Destruhaut^6-8^, Elise Arrive^1,2^, Christophe Bou^1-2^ ^1^CHU de Bordeaux, Pôle de Médecine et Chirurgie Bucco-Dentaire, 33000 Bordeaux, France. ^2^Université de Bordeaux, UFR des Sciences Odontologiques, 33076 Bordeaux, France. ^3^INSERM, Bio-ingénierie Tissulaire BioTisU1026, 33076 Bordeaux Cedex, France. ^4^CHU de Bordeaux, USMR, Pôle Santé publique, 33076 Bordeaux, France. ^5^CHU de Bordeaux, DRCI, 33076 Bordeaux, France. ^6^Université de Toulouse, Faculté de Chirurgie Dentaire,31062 Toulouse, France ^7^CHU de Toulouse, Hôpital de Rangueil, 31059 Toulouse Cedex, France ^8^Evolution and Oral Health Laboratory (EvolSan), Paul Sabatier University, Toulouse, France
Name and contact information for the trial sponsor {5b}	Patrick CASSAIDirection de la Recherche Clinique et de l’Innovation Promotion interne Responsable d’Etudes Cliniques Direction Générale des hôpitaux de Bordeaux 12 rue Dubernat 33404 TALENCE Cedex, FRANCE Tel : +33 (0)5 57 82 03 34 Fax : +33 (0)5 56 79 49 26 patrick.cassai@chu-bordeaux.fr
Role of sponsor {5c}	Through its employees, the sponsor roles are study design; collection, management, analysis, interpretation of data; writing of the report; and the decision to submit the report for publication

## Introduction

### Background and rationale {6a}

Acquired soft palate defects may be attributable to trauma, infection, or iatrogenic causes, but are usually caused by tumor excision or radiation necrosis. In 2018, the incidences of oropharyngeal cancer in France were 31.9 cases per 100,000 person-years for males and 10.9 cases per 100,000 person-years for females [1]. Soft palate tumors represent fewer than 15% of such cancers [2, 3]. Malignant soft palate tumors are commonly squamous cell carcinomas and are treated via chemoradiotherapy, radiotherapy, and/or surgery [4, 5]. Soft palate defects trigger velopharyngeal insufficiency associated with airflow escape, affecting swallowing (thus creating dysphagia) and speech (the vocal quality becomes hypernasal and intelligibility is compromised) [6–8]. These functional consequences impair the quality of life in both psychological and social terms.

The traditional approaches toward repair of soft palate defects include surgical reconstruction via flap placement and prosthetic rehabilitation with obturators, both combined with speech therapy [9]. Surgical reconstruction requires local, regional, or free flaps (depending on defect size) and affords definitive reconstruction [10, 11]. However, the limitations include a long hospital stay, possible donor-site morbidity, and risks associated with older age, systemic disease, radiotherapy, and/or the lack of adequate recipient vessels. A prosthetic pharyngeal obturator is a removable alternative allowing visual surveillance of cancer recurrence at low cost. The removable rigid prosthesis, designed in the nineteenth century, features a posterior extension that separates the oropharynx from the nasopharynx, efficiently restoring the sphincter when retention and stability are adequate [7]. A major limitation develops when the residual velopharyngeal muscles cannot contract to an extent permitting air passage modulation [8]. Such failure creates either a blockage or free space between the tissues and the obturator. Under such circumstances, the device does not fully restore phonation or swallowing function. The psychological and social repercussions compromise the quality of life. Optimal restoration of chewing and swallowing in such patients remains of great concern [12].

A recent case report described a membrane obturator prosthesis incorporating a dental dam that restored the soft palate, sealed the oropharynx during swallowing, and controlled nasal airflow during speech [13]. The rigid resin extension did not seal the opening, rather supporting a peripheral membrane that served as a valve that contacted residual tissue. Although the use of such a flexible obturator to treat soft palate defects seems promising, further clinical studies are required. We hypothesized that mimetic restoration of the velar anatomy using a membrane obturator (rather than a conventional rigid obturator) would be more likely to restore speech and swallowing functions and improve the quality of life of patients with acquired soft palate defects.

### Objectives {7}

Our principal objective in this random, crossover clinical trial is to compare the speech handicaps of patients with velar insufficiencies evident after surgery, and who were indicated for obturator treatment. The comparisons will be performed after patients wear either the new membrane obturator or a conventional rigid obturator for 1 month (in random order), employing the overall Voice Handicap Index (VHI) score. Our secondary objectives are to compare the swallowing status based on the global Deglutition Handicap Index (DHI) score and the health-related quality of life as revealed by answers to the questions posed by various domains of the EORTC QLQ-C30 and QLQ-H&N35 questionnaires.

### Trial design {8}

The trial protocol conforms with the Consolidated Standards of Reporting Trials (CONSORT) Statement. The trial design and protocol adhere to the Standard Protocol Items of the Recommendations for Interventional Trials (SPIRIT) criteria; the SPIRIT Checklist is attached (Table S1).

The VELOMEMBRANE trial is a superiority, open-labeled, two-way, random, crossover clinical trial comparing the new prosthesis to the rigid obturator in adult patients with acquired soft palate loss. The patients will wear both prostheses for 1-month periods in a random order.

## Methods: participants, interventions, and outcomes

### Study setting {9}

The bicentric trial will be conducted in the dental departments of teaching hospitals in Bordeaux (Prosthodontics and Periodontics Department, Saint André Hospital, Bordeaux, France) and Toulouse (Prosthodontics Department, Rangueil Hospital, Toulouse, France). Participants will be recruited from adults consulting the participating dental departments.

### Eligibility criteria {10}

The inclusion criteria will be:

- Age over 18 years

- Acquired loss of velar or palatovelar substance (class I and II a-d maxillectomies; Brown [2010]) after tumor excision

- Indicated for rigid obturator prosthesis rehabilitation

- Possible dental rehabilitation using a removable prosthesis (retention and stability assured without the use of glue, an extent of mouth-opening allowing finger entry, adequate saliva production, and a level of dexterity allowing of prosthesis insertion, removal, and cleaning)

- Provision of written informed consent

- Ability to speak and read French

- Availability and a willingness to present for regular follow-up during the entire study period

The exclusion criteria will be any of the following:

- Allergy to acrylic resin

- Current radiotherapy or chemotherapy

- Scheduled velar surgery during the 3 months required for prosthesis testing and analyses

- Prior maxillectomy (orbital floor or total)

- Pregnancy or lactation

- Participation in another interventional study

- An inability to give written informed consent

### Who will take informed consent? {26a}

All participants will be told of, and given an information sheet including, the names and affiliations of the investigators, a plain-language description of the study (the reference and experimental interventions), study duration, their right to withdraw at any time without giving reasons, ethics committee approvals, and the personal data privacy guarantee. After a period of reflection, during the enrolment visit, the written informed consent will be taken by the investigators.

### Additional consent provisions for collection and use of participant data and biological specimens {26b}

N/A. Neither collection nor use of participant data and biological specimens.

## Interventions

### Explanation for the choice of comparators {6b}

The conventional device, a rigid obturator (reference), rarely fully satisfying in restoring oral functions, will be compared to a new device, a membrane obturator, mimicking the missing anatomical structures.

### Intervention description {11a}

**Fig. 1:**
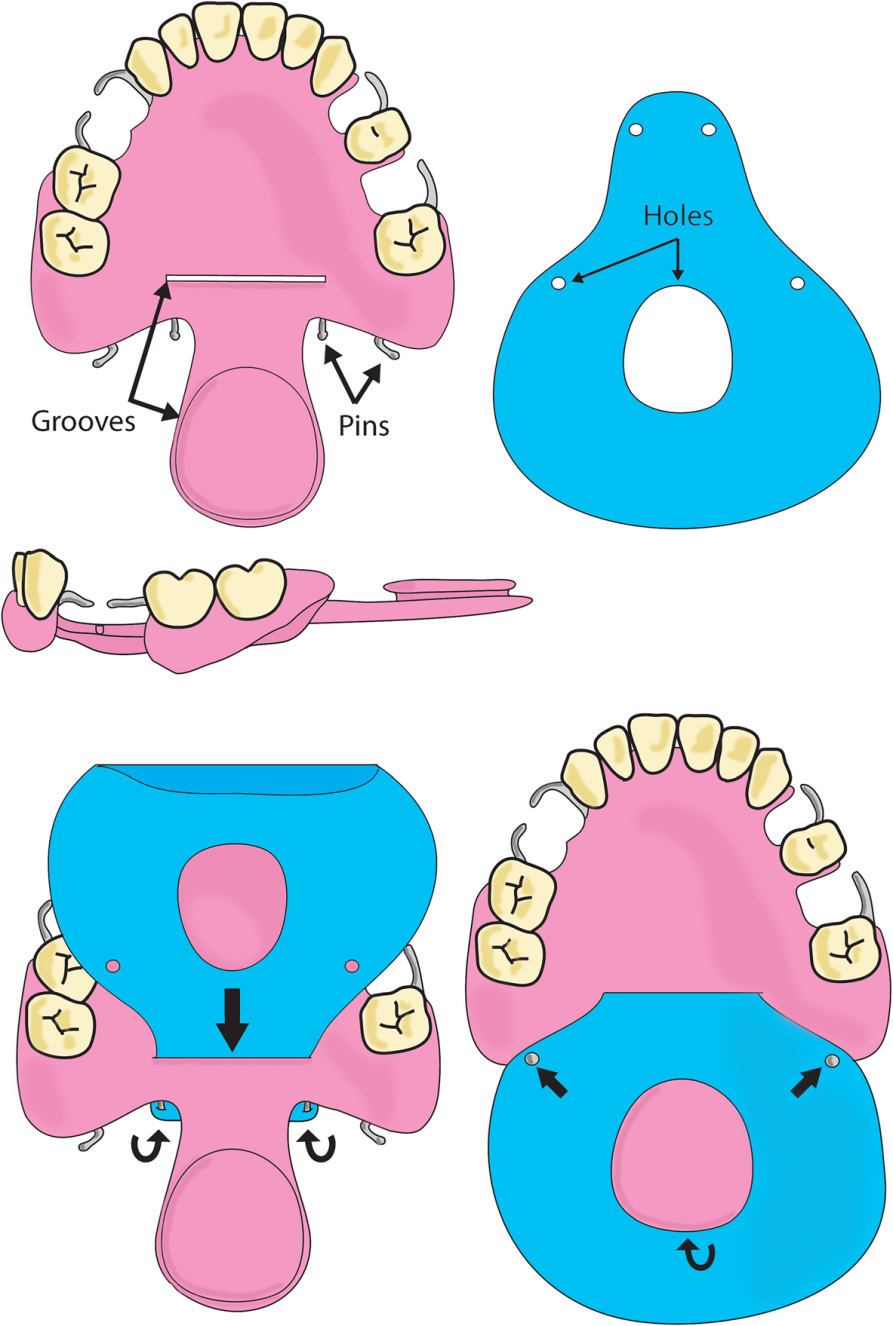
The membrane obturator prosthesis. **A** The device features a removable denture (or a palatal plate alone) with a rigid posterior extension (shown in the palatal and lateral views) and a thick dental dam. **B** The dental dam is inserted into the palatal groove and clipped onto medial denture pins. **C** Then the dam is pushed back, placed around the extension of the groove, and clipped onto exterior pins

**Fig. 2:**
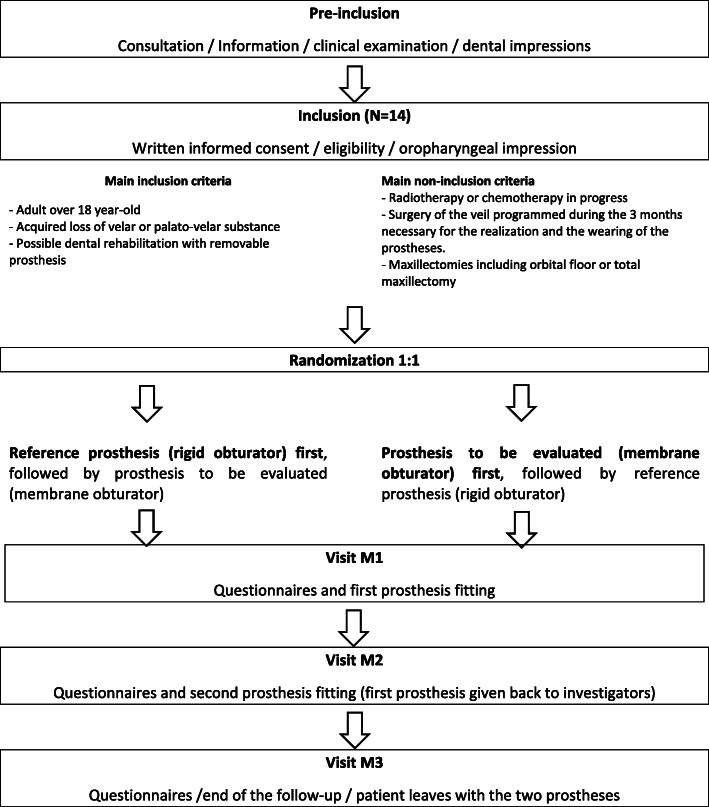
VELOMEMBRANE flowchart. The trial will evaluate the speech handicap in patients with acquired velar insufficiency at 1 month after wearing either the experimental membrane obturator or a conventional rigid obturator, based on the overall Voice Handicap Index (VHI) score, at the Bordeaux and Toulouse University Hospitals

We will obtain alginate impressions of the dental arches. Each provisional dental prosthesis created on these casts will include a posterior metal wire embedded in the palatal plate to support the oropharyngeal obturator fabricated from Kerr impression compound and lined with “SS White” impression paste. The plaster model will be duplicated in silicone. Thus, the same impression will be used to produce both the reference and experimental obturators (palatal plates) employing standard resin-handling procedures. For the reference rigid obturator, a rigid extension of acrylic resin will be added at the level of the hard palate that will allow surface contact with the remaining musculature. In the new membrane obturators, the rigid resin extension will be positioned in the soft palate plane prior to removal from the mold. The borders will be trimmed to lie 5 mm distant from the (contracted) remaining musculature. Pins and grooves will be added to ensure membrane retention (Fig. 1). A thick dental dam (Dental Hygienic Corporation) will serve as the membrane and will be shaped to ensure a 10-mm overlap with the pharyngeal walls, effectively forming an artificial soft palate [13]. Each patient will wear each obturator for 1 month, in random order. A trial flowchart is shown in Fig. 2.

### Criteria for discontinuing or modifying allocated interventions {11b}

If a participant declares not using the study obturator, he/she will be considered as a drop-out at the time of the study visit.

### Strategies to improve adherence to interventions {11c}

During the study, participants will have only the obturator to be used in the month in question. At each study visit, they will be asked if and how they use the obturator.

### Relevant concomitant care permitted or prohibited during the trial {11d}

Chemotherapy, radiotherapy, and maxillofacial surgery are prohibited during the trial as these treatments can affect the oral functions (oral pain, intolerance to prosthesis, loss of prosthetic adaptation, etc.).

### Provisions for post-trial care {30}

At the end of the study, patients will be proposed to continue their long-term oral follow-up at the Dental department of the Bordeaux or Toulouse teaching hospital.

### Outcomes {12}

The primary outcome will be phonation-related disability based on the overall score of the Voice Handicap Index (VHI) [14, 15] at 1 month after delivery of either prosthesis. This ranges from 0 and 120; the VHI includes 30 items exploring functional, emotional, and physical domains. The secondary outcomes include the Deglutition Handicap Index (DHI, 30 items, maximum score 120) and the EORTC QLQ-C30 (a general questionnaire containing the QLQ-H&N35 modules [35 items in all] exploring the health-related quality of life) prepared by the European Organization for Research and Treatment of Cancer (EORTC) [16–18]. Both questionnaires are scored from 0 to 100 and will be evaluated at 1 month after the delivery of either prosthesis. All questionnaires have been translated into French and validated.

### Participant timeline {13}

The study timeline is summarized in Table 1. Patients will be included over 21 months, and the trial will run for 3 months, creating a final duration of study of 24 months.

**Table 1:** VELOMEMBRANE study timeline: enrollment, interventions, and assessments

		Study time			
		Enrolment and allocation	Post-allocation		Close-out
Timepoints	−t_1_	0	M1 30 days	M2 60 days	M3 90 days
Enrollment					
Clinical examination	✓				
Collection of dental arch Impressions	✓				
Occlusion examinations	✓				
Eligibility screening	✓				
Collection of information	✓				
Obtaining informed consent		✓			
Allocation to groups		✓			
Collection of velopharyngeal impressions		✓			
Interventions					
Fitting of obturator prostheses			✓	✓	
Assessments					
Questionnaire surveys			✓	✓	✓

### Sample size {14}

Sample size calculation is based on the average comparison of crossover tests: the *t*-test for difference of means in 2 × 2 crossover design (N-Query software). We assume that the use of the rigid obturator will be associated with a mean VHI score of 34 and a standard deviation of 15 [14, 15]. To ensure a power of 90%, an alpha risk of 5%, and an expected difference of 15 points between the reference and new obturator, 7 patients per treatment sequence are required, thus 14 in all. No drop-out was included in the sample size calculation due to the small duration of the study follow-up (3 months).

### Recruitment {15}

For achieving adequate participant enrolment to reach the target sample size, all new patients meeting eligibility criteria will be given the opportunity to participate in the study. A close coordination is made between the Dental departments and the Oncology and Maxilla-facial surgery departments of both teaching hospitals to identify and refer adequately potential participants.

## Assignment of interventions: allocation

### Sequence generation {16a}

Assignments will be prepared by the statistician of the Bordeaux University Hospital Clinical Trial Unit (CTU) prior to trial commencement, using SAS ver. 9.4 software (SAS Institute Inc., Cary, NC, USA). Randomization is balanced (1:1 ratio) in random permuted blocks and stratified on the site.

### Concealment mechanism {16b}

The secure CTU website will contain data on patient eligibility, allocation numbers, and randomization group. The CTU statistician and computer scientist are the only ones with access to the randomization list.

### Implementation {16c}

The randomization list is implemented in the RedCap software by the study statistician. Access to the randomization list via the e-CRF is limited to the study investigators.

## Assignment of interventions: blinding

### Who will be blinded {17a}

N/A. Neither investigator nor patient will be blinded.

### Procedure for unblinding if needed {17b}

N/A. Not applicable.

## Data collection and management

### Plans for assessment and collection of outcomes {18a}

The VHI questionnaire will be administered by the investigator at baseline and 1 month after the delivery of either prosthesis. The secondary outcomes will be obtained from self-administered questionnaires completed at baseline and 1 month after each device is tested. Patient’s answers will be entered in e-CRF through a dedicated REDCap entry mask.

### Plans to promote participant retention and complete follow-up {18b}

A patient will be considered as lost-to-follow-up if no contact can be made during 3 months, after an active search from the investigator. Participants will receive no financial compensation but they will be given both obturators at the end of the study.

### Data management {19}

Data are entered anonymously in e-CRF through a dedicated REDCap entry mask. Data collection will be monitored by a clinical research assistant. When requested, the investigator will clarify data. Data management is under the responsibility of the Bordeaux University Hospital CTU.

### Confidentiality {27}

The final trial dataset will be available to the clinical research assistants, data managers, and statisticians, subject to professional secrecy. Data are anonymous.

### Plans for collection, laboratory evaluation, and storage of biological specimens for genetic or molecular analysis in this trial/future use {33}

N/A. No collection.

## Statistical methods

### Statistical methods for primary and secondary outcomes {20a}

Scores will be evaluated in terms of mean, standard deviation, median, range, and first and third quartiles. To assess the results, a mixed-effects linear regression model will be used to analyze effects of “treatment” and “period.” Analyses will vary by whether an interaction between treatment and period is or is not apparent. The response variable of the linear regression model will be the overall VHI score; the explanatory variables will be the treatment group (a fixed effect), the administration period (a fixed effect), the interaction between treatment and period (a fixed effect), and the patient (a random effect). The same statistical methods will be used for the secondary outcomes.

Sensitivity analyses will be performed by adding “interaction treatment*period.”

### Interim analyses {21b}

N/A. No interim analyses are planned.

### Methods for additional analyses (e.g., subgroup analyses) {20b}

N/A. No additional analyses are planned.

### Methods in analysis to handle protocol non-adherence and any statistical methods to handle missing data {20c}

Protocol violations after randomization will be listed in the Clinical Study Report, tabulated by subject and recruitment site. We will perform intention-to-treat analyses with a “missing=failure” strategy to the management of missing data.

Sensitivity analyses will be performed for the missing data management: multiple imputation, available data, and maximum bias.

### Plans to give access to the full protocol, participant-level data, and statistical code {31c}

The full protocol, participant-level data, and statistical code are available upon request and after a contract has been put in place to ensure, among other things, that the recipient complies with the GDPR.

## Oversight and monitoring

### Composition of the coordinating center and trial steering committee {5d}

The steering committee is made up of the following people: Dr Adrien NAVEAU (chairman, principal investigator), Dr Christophe BOU (clinical investigator and responsible for the obturator manufacturing), Dr Florent DESTRUHAUT (clinical investigator and responsible for the center of Toulouse), Dr Elise ARRIVE (methodologist), a statistician, Dr Emilie RENARD (pharmacist), and a representative of the sponsor. This committee checks ethics. With the Center for Methodology and Data Management, this committee checks also the status of the research, possible problems, and available results. It decides on any relevant modification of the protocol necessary for the continuation of the research. It may propose to extend or interrupt the research.

### Composition of the data monitoring committee, its role, and reporting structure {21a}

The establishment of a data monitoring committee is not necessary for this study, which does not entail any particular risk a priori for the participants.

### Adverse event reporting and harms {22}

Adverse events that may occur will be monitored by the investigators and a research assistant. Possible adverse events include ingestion/inhalation of imprint materials, metal pins, resin, or prosthesis or membrane materials; oral injury; and allergies to acrylic resin, chrome-cobalt (of the metal pins), or latex.

### Frequency and plans for auditing trial conduct {23}

In the context of the data monitoring plan, a clinical research assistant mandated by the sponsor will visit each investigating center on a regular basis, during the implementation of the research, one or more times during the research according to the rhythm of the inclusions and at the end of the research. An audit can be conducted any time at the request of the sponsor and independent from the investigators, but also at the request of the competent health authority.

### Plans for communicating important protocol amendments to relevant parties (e.g., trial participants, ethical committees) {25}

Any important protocol amendment must obtain, prior to its implementation, a favorable opinion from a French Ethics Committee for Person Protection (Comité de Protection des Personnes [CPP]) and an authorization from the French National Agency for Drug Safety (Agence Nationale de Sécurité du Medicament et Produits de Santé [ANSM]). All modifications to the protocol should be brought to the attention of all investigators participating in the research. Any modification that modifies the coverage of participants or the benefits, risks, and constraints of the research is the subject of a new information sheet and a new consent form.

## Dissemination plans {31a}

Results of the trial will be communicated to the participants through a brochure that will be sent at the patient’s home. They will be also submitted to national and international journals for publication.

Results of the trial will be communicated to the participants upon request to the investigator.

## Discussion

Our hypothesis is that the new membrane obturator will improve speech more than the standard obturator [19]. The primary outcome is therefore the Voice Handicap Index (VHI) score. This dysphonia self-assessment scale quantifies the extent to which a phonation disorder compromises the quality of life and assesses the psychosocial consequences. The French version has been validated and used in patients with oropharyngeal cancer [14, 20]. The Deglutition Handicap Index (DHI) is a derivative of the VHI that focuses on swallowing [18]. This questionnaire quantifies the adverse effects of swallowing disorders and the score will serve as a secondary outcome.

Soft palate defects arise in various ways. However, we will include only patients who have undergone surgical tumor removal. Soft palate trauma is very rare. Patients with congenital defects such as palatal clefts usually develop mechanisms that compensate for speech and swallowing difficulties; these become entrenched and are difficult to bypass. Oncology patients should be able to adapt to the use of a new device; our new prosthesis should restore speech and improve swallowing, improving the quality of life [19].

“Quality of life” includes physical components (autonomy and physical activities), psychological components (anxiety, depression, and emotion), relational components (family, social, and professional), symptomatic components (repercussions of the disease and treatment thereof), and other components (sexuality and self-image). The relevant psychometric tools (scales or questionnaires) are easy to use and well standardized. The QLQ-H&N35/EORTC QLQ-C30 questionnaires of the EORTC are commonly used for ear-nose-and-throat assessments [16, 17, 21, 22]. French versions have been employed to evaluate patients with oropharyngeal cancers [21, 23, 24]. Both questionnaires yield several scores (thus not single overall scores) and therefore cannot serve as principal assessment criteria. In addition, they explore aspects of the quality of life that are not related to our intervention. We will perform secondary analyses only.

The crossover design is optimal for several reasons:

- The two prostheses are removable and their effects do not persist. Thus, the initial state is re-attained at the beginning of the second period; no “washout” is required
- There is no further substance loss over the test period
- The risk of loss to follow-up is minimal; the test periods are short (1 month)
- Outcomes of interest will be measured at 1-month intervals; no conditioning effect is in play

This study will yield high-quality evidence on the utility of a new device that should greatly improve the management and the quality of life of patients with acquired velar insufficiencies. Such insufficiencies cause malnutrition, reduce the quality of life, can lead to patient refusal to accept care, and may trigger psychological and social isolation associated with poor communication and societal integration [8, 25]. Often, the rigid obturator does not restore oronasal partitioning; the patient remains disabled. The membrane obturator will remedy swallowing and phonation deficiencies more effectively and rapidly than a rigid obturator.

## Trial status

This trial is registered as CHUBX 2018/34. This report is based on protocol version 1.0. Recruiting has started in July 2020 and continues for 21 months. Due to the COVID-19 crisis, the initial deadline of April 2022 may be delayed of few additional months (to be determined).

## Supplementary Information

**Additional file 1: Table S1**: SPIRIT 2013 Checklist.

## Abbreviations

ANSM: Agence Nationale pour la Sécurité du Médicament et des Produits de Santé ([French] National Agency for Medicines and Health Products Safety)
CONSORT: Consolidated Standards of Reporting Trials
CPP: Comité de Protection de Personnes ([French] Ethics Committee for the Protection of Persons)
CRF: Case report form
CHU: Centre Hospitalo-Universitaire (University Hospital Center)
CTU: Bordeaux University Hospital Clinical Trial Unit
